# Microstructure and gene expression influence gyrification in amyotrophic lateral sclerosis

**DOI:** 10.1093/braincomms/fcaf491

**Published:** 2025-12-16

**Authors:** Yihan Jiang, Yan Fu, Xinyu Song, Xueying Wang, Jianyu Li, Luqi Cheng, Yifan Chen, Yuanchao Zhang, Junling Wang, Xiaoping Yi, Lena Palaniyappan

**Affiliations:** The Clinical Hospital of Chengdu Brain Science Institute, MOE Key Lab for Neuroinformation, University of Electronic Science and Technology of China, Chengdu 610054, P. R. China; School of Life Science and Technology, University of Electronic Science and Technology of China, Chengdu 610054, China; Department of Radiology, Tongji Hospital, Tongji Medical College, Huazhong University of Science and Technology, Wuhan 430030, P.R. China; Xiangya Hospital, Central South University, Jiangxi (National Regional Center for Neurological Diseases), Nanchang, Jiangxi 330038, P. R. China; Department of Radiology, Xiangya Hospital, Central South University, Changsha , Hunan 410008, China; School of Life Science and Technology, University of Electronic Science and Technology of China, Chengdu 610054, China; School of Life and Environmental Sciences, Guilin University of Electronic Technology, Guilin 541004, China; School of Life Science and Technology, University of Electronic Science and Technology of China, Chengdu 610054, China; The Clinical Hospital of Chengdu Brain Science Institute, MOE Key Lab for Neuroinformation, University of Electronic Science and Technology of China, Chengdu 610054, P. R. China; School of Life Science and Technology, University of Electronic Science and Technology of China, Chengdu 610054, China; College of Health Solutions, Arizona State University, Phoenix, AZ 85004, USA; Xiangya Hospital, Central South University, Jiangxi (National Regional Center for Neurological Diseases), Nanchang, Jiangxi 330038, P. R. China; Hunan International Scientific and Technological Cooperation Base of Neurodegenerative and Neurogenetic Diseases, Changsha, Hunan 410008, P. R. China; National Clinical Research Center for Geriatric Diseases, Xiangya Hospital, Central South University, Changsha, Hunan 410008, P. R. China; Key Laboratory of Hunan Province in Neurodegenerative Disorders, Central South University, Changsha, Hunan 410008, P. R. China; Center for Medical Genetics, School of Life Sciences, Central South University, Changsha, Hunan 410008, P. R. China; Engineering Research Center of Hunan Province in Cognitive Impairment Disorders, Central South University, Changsha, Hunan 410008, P. R. China; Department of Radiology, Chongqing University Three Gorges Hospital, Chongqing University, Chongqing 410008, P.R. China; Clinical Research Center (CRC), Medical Pathology Center (MPC), Cancer Early Detection and Treatment Center (CEDTC) and Translational Medicine Research Center (TMRC), Chongqing University Three Gorges Hospital, Chongqing University, Chongqing 404000, P.R. China; School of Medicine, Chongqing University, Chongqing 400030, P.R. China; Douglas Mental Health University Institute, McGill University, Montreal, Quebec, Canada, H4H 1R3

**Keywords:** amyotrophic lateral sclerosis, cortical gyrification, fractional anisotropy, transcription

## Abstract

Amyotrophic lateral sclerosis is a fatal neurodegenerative disease involving progressive degeneration of upper and lower motor neurons. Beyond well-established grey and white matter pathology, alterations in cortical gyrification have recently been observed, yet their clinical relevance and molecular underpinnings remain to be understood. Here, we investigated this premise by examining its microstructural and transcriptional basis in 60 patients with amyotrophic lateral sclerosis (median age = 55, range = 25–72 years) and 60 matched controls (median age = 56, range = 27–72 years) using structural and diffusion MRI. Patients exhibited a significant reduction in local gyrification index within bilateral precentral and postcentral gyri, left middle frontal gyrus and left superior parietal lobule. This was accompanied by reduced fractional anisotropy in the white matter tracts, primarily involving the corticospinal tract and corpus callosum. Higher local gyrification index and fractional anisotropy values were associated with better motor function as measured by the Amyotrophic Lateral Sclerosis Functional Rating Scale-Revised, and local gyrification index also showed positive associations with global cognitive status. A mediation analysis indicated that fractional anisotropy partially accounted for the relationship between local gyrification index and functional disability, suggesting that disrupted white matter pathways contribute to the clinical impact of gyrification changes. To explore underlying mechanisms, we integrated neuroimaging findings with transcriptomic data from the Allen Human Brain Atlas. Regions of reduced local gyrification index showed spatial convergence with cortical expression of amyotrophic lateral sclerosis-related genes such as *TARDBP* and *C9orf72*, enriched for biological processes related to protein aggregation, axon guidance and synaptic signalling. Together, these findings suggest that cortical gyrification abnormalities in amyotrophic lateral sclerosis are closely linked to white matter degeneration, functional impairment and genetic vulnerability, thereby offering an integrative window into the multiscale pathology of amyotrophic lateral sclerosis.

## Teaser

Jiang *et al*. report that reduced cortical gyrification in amyotrophic lateral sclerosis is associated with white matter tract degeneration and clinical disability. Furthermore, these cortical folding abnormalities are spatially correlated with the expression of amyotrophic lateral sclerosis-related genes, linking macroscopic anatomical changes to their underlying transcriptional signatures.

## Graphical Abstract

**Graphical Abstract:**
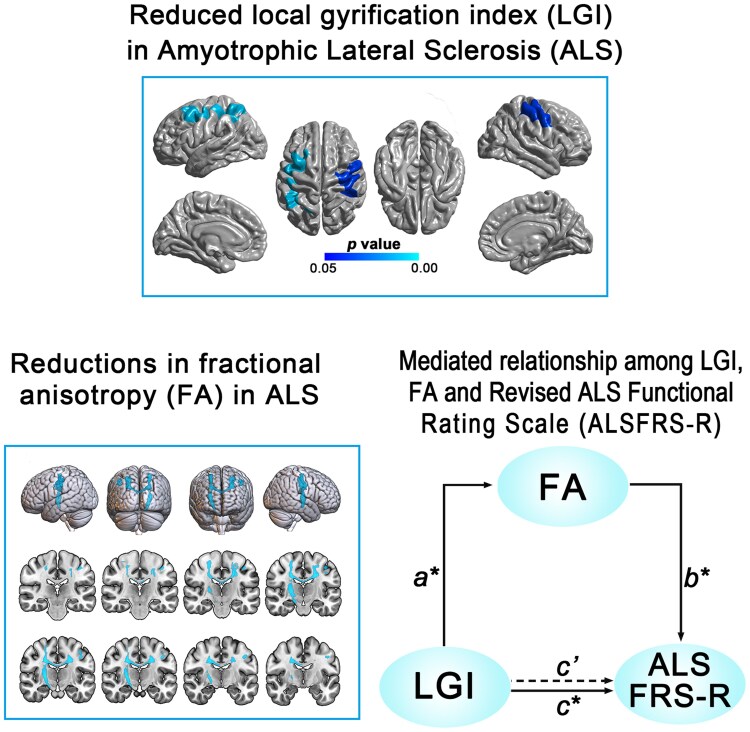

## Introduction

Amyotrophic lateral sclerosis (ALS) is a fatal neurodegenerative disease characterized clinically by spasticity, hyperreflexia, muscle weakness, dysphagia and respiratory failure, ultimately resulting in death within approximately 3–5 years from the onset of symptoms.^1,2^ The motor symptoms of ALS have been predominantly attributed to the degeneration and loss of both the upper motor neurons of the corticospinal tract, and lower motor neurons of the brainstem and anterior horn of the spinal cord.^3,4^ Histopathologically, the neurodegenerative process of ALS appears to track the formation of intracellular aggregates consisting mainly of phosphorylated 43-kDA TAR DNA-binding protein (pTDP-43).^3,5,6^ Based on post-mortem samples of patients with ALS, Braak *et al*., revealed a stereotypical distribution of pathogenic pTDP-43 aggregates,^7,8^ which propagate from cell to cell along axonal projections.^9,10^ By simulating the sequential dissemination of pTDP-43 using connectome-based analyses,^9-11^ neuroimaging studies provide further *in vivo* evidence to support this hypothesis.

One of the most prominent morphologic characteristics of human brain is its folding (gyrification); this allows a much larger cortical surface area to fit inside the skull.^12^ As this folded contour critically depends on the optimized organization of underlying axonal connections, even subtle disruptions in white matter integrity affect the gyrification of connected regions.^13,14^ Cortical gyrification can be readily measured using a single short-duration anatomical MRI; thus, quantitative measure of gyrification may serve as a surrogate marker of the underlying microstructural abnormalities in white matter tracts.^15-17^ To date, only a limited number of studies have examined the gyrification pattern in ALS, but all of them support the potential utility as an early marker. For instance, individuals with a higher genetic risk for ALS (i.e. presymptomatic *C9orf72* expansion carriers) have reduced gyrification in precentral gyrus, inferior parietal lobule, superior occipital gyrus, inferior and middle frontal gyrus, among others.^18,19^ Compared with healthy controls, significantly reduced gyrification in the right occipital cortex is reported in a sample of 25 symptomatic patients with ALS.^20^ However, we do not yet know if these gyrification changes in ALS are linked to white matter microstructural abnormalities representing degenerative changes in the corticospinal tract and corpus callosum. If this is the case, we can expect gyrification changes to be associated with the degree of axonal damage, and to be spatially distributed in regions where preferential gene expression relevant to the molecular pathways of pTDP-43 aggregation occurs. Studies as such may contribute to a better understanding of the pathophysiological mechanisms of this disorder.

In the present study, we build on the existing literature on the pathophysiology of ALS,^18,20,21^ to test whether (i) patients with ALS show significantly reduced cortical gyrification measured by local gyrification index (LGI) in regions critically involved in motor functioning compared with healthy controls (HCs); (ii) fractional anisotropy (FA) is significantly reduced in the whiter matter tracts connecting the regions with atypical gyrification in ALS; (iii) gyrification carries prognostic information regarding functional abilities in ALS, and if the white matter microstructure mediates the relationship between gyrification and functioning; and (iv) if the distribution of atypical gyrification in ALS relates to the molecular pathways critical for the formation and propagation of pTDP-43 aggregates along axonal pathways. Considering the progressive nature of ALS,^22^ we aim to provide early empirical evidence to support the potential use of repeated MRI-based gyrification assessment to track the clinical course of ALS in individuals affected by or predisposed to ALS.

## Materials and methods

### Participants

A total of 60 patients with ALS (28 women, 32 men, median age = 55 years, range = 25–72 years, right-handed) and 60 demographically matched HCs (26 women, 34 men, median age = 56 years, range = 27–72 years, right-handed) were included in the present study. Specifically, patients were consecutively recruited from the Department of Neurology of Xiangya Hospital, Central South University, Changsha, China. All patients were diagnosed with sporadic probable or definite ALS using the revised El Escorial criteria^23^ by experienced neurologists. All participants underwent standardized neurological and neuropsychological examinations on the day of magnetic resonance scanning. Individuals were excluded if they had a family history of any neurological disorder; or any other major systemic, psychiatric or neurologic illnesses, such as epilepsy, stroke or structural brain abnormalities; and other causes of focal or diffuse brain damage, including lacunae and extensive cerebrovascular disorders at routine MRI. Details of the recruitment process for this cohort can be found in the flowchart (Supplementary Fig. 1).

Demographic, clinical, laboratory, treatment and outcome data were all prospectively collected using a standardized data collection form. All patients were newly diagnosed, and had not received any systemic therapy for this disease. All patients were evaluated using the Revised Amyotrophic Lateral Sclerosis Functional Rating Scale (ALSFRS-R) for motor impairments, the King’s College Staging system for clinical staging, the Mini-mental State Examination (MMSE) for cognitive functioning, the Hamilton Depression Scale (HAMD) for depression, the Hamilton Anxiety Scale (HAMA) for anxiety and the Fatigue Severity Scale (FSS) for fatigue. The disease duration (i.e. from symptom onset to study enrollment) was also recorded for each patient. All clinical and neuropsychological and behavioral data were evaluated by two physicians (L.W. and J.D.) and a third senior researcher (J.W.) adjudicated any differences in interpretation between the two primary reviewers.

The study was approved by the Ethics Committee and the Expert Committee of Xiangya Hospital, Central South University (IRB#, 20201120425), and written informed consent was obtained from all participants.

### MRI data acquisition

The MRI data of patients with ALS and matched HCs were collected on a Siemens MAGNETOM Prisma 3.0 T MRI scanner with a 64-channel head coil at the Department of Radiology, Xiangya Hospital, Central South University. The neuroimaging protocol consisted of T1-weighted and diffusion-weighted images. For each participant, the T1-weighted images were acquired using a standard magnetization prepared rapid 3D gradient-echo sequence with 208 sagittal slices (repetition time [TR] = 2300 ms; echo time [TE] = 2.98 ms; inversion time = 900 ms; flip angle = 9°; matrix size = 256 × 256 mm^2^; slice thickness = 1.0 mm; voxel size = 1 × 1 × 1 mm^3^; no slice gap). The diffusion-weighted images (*b*-values: 0, 1000 and 2000 s/mm^2^; 128 diffusion directions) were acquired using an echo planar imaging sequence with 70 axial slices (TR = 4300 ms; TE = 75 ms; flip angle = 90°; matrix size = 132 × 132; slice thickness = 2 mm; no slice gap). To minimize motion artifacts, all subjects were instructed to stay awake and not to fall asleep during scanning.

To obtain an unbiased estimation of the white matter tracts connected to regions with atypical gyrification using probabilistic tractography, high spatial and angular resolution multi-modal MRI data of 40 healthy adults (20 male; ages: 22–36 years) selected from the S1200 subjects release of the Human Connectome Project database (HCP; http://www.humanconnectome.org/study/hcp-young-adult/) were included in this study.^24^ The detailed imaging protocol can be found at https://www.humanconnectome.org/documentation/Q1/imagingprotocols.html. Briefly, T1-weighted images (field of view: 224 × 320; slices: 256; flip angle: 8°; resolution: 0.7 mm isotropic), and diffusion-weighted images (field of view: 210 × 180; slices: 111; flip angle: 78°; resolution: 1.25 mm isotropic; *b*-values: 1000, 2000 and 3000 s/mm^2^) were collected on a 3.0 T Skyra scanner (Siemens, Erlangen, Germany) using a 32-channel head coil.

### Cortical gyrification analysis

In this study, cortical gyrification was quantitatively assessed using Schaer’s LGI,^25^ which is a three-dimensional vertex-wise extension of the classical gyrification index.^26^ The LGI map of each participant can be generated from T1-weighted images using FreeSurfer (http://surfer.nmr.mgh.harvard.edu/) through four sequential steps: First, the T1-weighted images were preprocessed to reconstruct the pial surface in three-dimensional space. Second, an artificial outer surface representing the convex hull of the pial surface was created. Third, the LGI for a given vertex on the outer surface was computed as a ratio between the area of two correspondent surface patches generated respectively on the pial and outer surfaces. Fourth, the LGI values of the outer surface were reallocated to the vertices of the pial surface considering their prior contributions to the computation of relevant outer-surface LGI values. After that, the LGI map was resampled to the FreeSurfer fsaverage template and subsequently smoothed with a 15-mm full-width-at-half-maximum (FWHM) Gaussian kernel.

Vertex-wise contrast of LGI was done between patients with ALS and HCs using SurfStat package (http://www.math.mcgill.ca/keith/surfstat/). A general linear model (GLM) was fitted independently at each vertex of the cortical surface in both hemispheres, with age, sex and group included as covariates. Candidate clusters were first identified using a vertex-level threshold of *P* < 0.01. Random field theory (RFT) was then applied at the cluster level across the entire cortical surface to correct for multiple comparisons. Clusters that survived this correction were considered statistically significant at *P* < 0.05.

### Voxel-wise FA analysis

Diffusion-weighted images of the patients with ALS and HCs were processed with the FMRIB Software Library (FSL; https://fsl.fmrib.ox.ac.uk/fsl/). Initially, images were corrected for eddy current using affine registration to minimize the effect of head movement. After eddy correction, the FSL Brain Extraction Tool was used to extract the brain and exclude non-brain tissues such as the skull and scalp. Subsequently, the FA map was acquired by fitting a diffusion tensor model to each voxel, and was registered to the Montreal Neurological Institute (MNI) space by applying the linear and nonlinear registration tools provided by FSL. Further, before conducting statistical analysis, the FA maps were smoothed with a FWHM of 8 mm isotropic Gaussian kernel.

T1-weighted and diffusion-weighted images of the 40 participants of the HCP dataset were preprocessed with the HCP’s minimal preprocessing pipeline.^27^ After preprocessing, voxel-wise estimates of the fibre orientation distribution of diffusion-weighted images were calculated using FSL’s Bedpostx, allowing for three fibre orientations. To reconstruct the white matter tracts connected to regions with atypical gyrification, probabilistic tractography was performed with FSL’s Probtrackx and seeded from each voxel in the regions with atypical gyrification to the whole brain by drawing 5000 streamline samples. To eliminate spurious connections, the raw streamline counts for each subject were thresholded at a streamline count of ≥1. The resulting volume of tracts was binarized and registered to MNI space. A population tractogram was obtained by calculating a probabilistic fibre-tract map across subjects and thresholding it at 50% probability.

Voxel-wise contrast of FA was done between patients with ALS and HCs using the Data Processing & Analysis of Brain Imaging (DPABI) (http://rfmri.org/dpabi) package. The GLM used in the vertex-wise LGI analysis was repeated to test for significant FA differences between the two groups. By applying the aforementioned population tractogram as an explicit mask, the voxel-wise FA analysis was constrained to the white matter tracts connected to the regions with atypical gyrification. Clusters of interest were firstly defined using a vertex-wise *P* < 0.001 and then corrected for multiple comparisons using Gaussian random field theory (GRFT). The significance level was set at GRFT-corrected *P* < 0.05.

### Correlation analyses between imaging and clinical data

In the patient group, Pearson’s correlation coefficient was used to explore the relationship between the mean LGI/FA of the significant clusters and relevant clinical variables including ALSFRS-R score, MMSE, among others. The level of significance was set at *P* < 0.05.

### Intermodal correlation analysis

Pearson’s correlation coefficient was used to explore the relationship between the mean LGI of the affected cortical regions and mean FA of the affected white matter tracts, respectively, for patients with ALS and healthy controls. The level of significance was set at *P* < 0.05.

### Mediation analyses

To explore the relationship among LGI, FA and ALSFRS-R score in patients with ALS, two separate mediation analyses were conducted using Mediation Toolbox^28^ in MATLAB. In the first mediation analysis, we tested if the mean FA of the affected white matter tracts mediates the relationship between the mean LGI of the affected cortical regions and ALSFRS-R score. In the second mediation analysis, we tested if the mean LGI of the affected cortical regions mediates the relationship between the mean FA of the affected white matter tracts and ALSFRS-R score.

### Neuroimaging–transcription association analysis

To investigate the molecular mechanisms potentially underlying cortical gyrification changes in ALS, we conducted a neuroimaging–transcriptomic association analysis. For each of the 68 cortical regions defined by the Desikan–Killiany Atlas,^29^ the mean *t*-value was extracted from the vertex-wise LGI contrast map, producing a 68 × 1 vector representing regional gyrification deviations. Gene expression data were obtained from the Allen Human Brain Atlas and mapped onto the same cortical regions using the abagen toolbox (https://github.com/rmarkello/abagen), resulting in a 68 × 16 533 regional transcription matrix. Partial least squares (PLS) regression was applied to assess the relationship between regional gene expression and gyrification deviations, with the transcription matrix as predictors and LGI deviations as the response variable. PLS identifies latent components that maximize the covariance between predictors and responses,^28,29^ with the first component (PLS1) representing the linear combination of gene expression values that best accounts for regional LGI differences. The significance of PLS1 was evaluated using 5000 permutations. To estimate the reliability of each gene’s contribution, bootstrap resampling of cortical regions was performed 1000 times, and *Z*-scores were calculated by dividing each gene’s PLS1 weight by its bootstrap-derived standard error. Only genes with a *Z*-score > 3 or *Z*-score < −3 were considered for enrichment analysis. Functional enrichment of these genes was analysed using Metascape (https://metascape.org/) to identify relevant Gene Ontology (GO) biological processes and Kyoto Encyclopaedia of Genes and Genomes (KEGG) pathways.^30^

### Statistical analysis

Group differences in demographic and clinical characteristics between patients and HCs were analysed using SPSS. Continuous variables (age, MMSE, HAMD, HAMA and FSS scores) were compared with independent-samples *t*-tests, while categorical variables (sex distribution) were assessed with Chi-square tests. The significance level was set at *P* < 0.05 (two-tailed).

## Results

### Demographic and clinical characteristics

The demographic and clinical data of all participants are presented in Table 1. Of the 60 patients with ALS, 50 had a limb onset (83.3%) and 9 had a bulbar onset (15.0%). The median disease duration for the patient group was 12.00 months (IQR: 8.00–21.00). According to the King’s College Staging system, 26 (43.3%) patients were in Stage 1, 19 (31.7%) in Stage 2 and 15 (25.0%) in Stage 3. There were no significant differences in age (*t* = −1.522, *P* = 0.131) or sex (χ^2^ = 0.034, *P* = 0.854) between patients with ALS and HCs. Relative to HCs, patients with ALS had significantly higher scores on the MMSE (*t* = 3.978, *P* < 0.001), HAMD (*t* = −3.548, *P* < 0.001), HAMA (*t* = −2.957, *P* = 0.004) and FSS (*t* = −7.926, *P* < 0.001).

**Table 1:** Demographic and clinical data of patients with ALS and healthy controls

	Patients with ALS (*N* = 60)	Healthy controls (*N* = 60)	*P*-value
Age, years	56.0 (48.00–62.75)	55.00 (45.75–58.00)	0.131
Male/female	32/28	34/26	0.854
Limb/bulbar/both onset	50/9/1		
King’s College Stage (1/2/3/4)	26/19/15/0		
Disease Duration (months)	12.00 (8.00–21.00)		
ALSFRS-R score	40.00 (37.75–45.00)		
MMSE score	28.00 (26.00–30.00)	29.00 (28.00–30.00)	<0.001
HAMD score	4.00 (2.50–8.50)	3.00 (1.75–5.00)	<0.001
HAMA score	5.00 (2.50–10.00)	4.00 (2.00–6.00)	0.004
FSS score	20.00 (12.25–33.00)	9.00 (9.00–12.00)	<0.001

ALS, amyotrophic lateral sclerosis; ALSFRS-R, ALS Functional Rating Scale Revised; MMSE, mini-mental state examination; HAMD, Hamilton Depression Scale; HAMA, Hamilton Anxiety Scale; FSS, fatigue severity scale. All continuous variables were stated as median, 25th and 75th percentile.

### Cortical gyrification analysis

**Figure 1:**
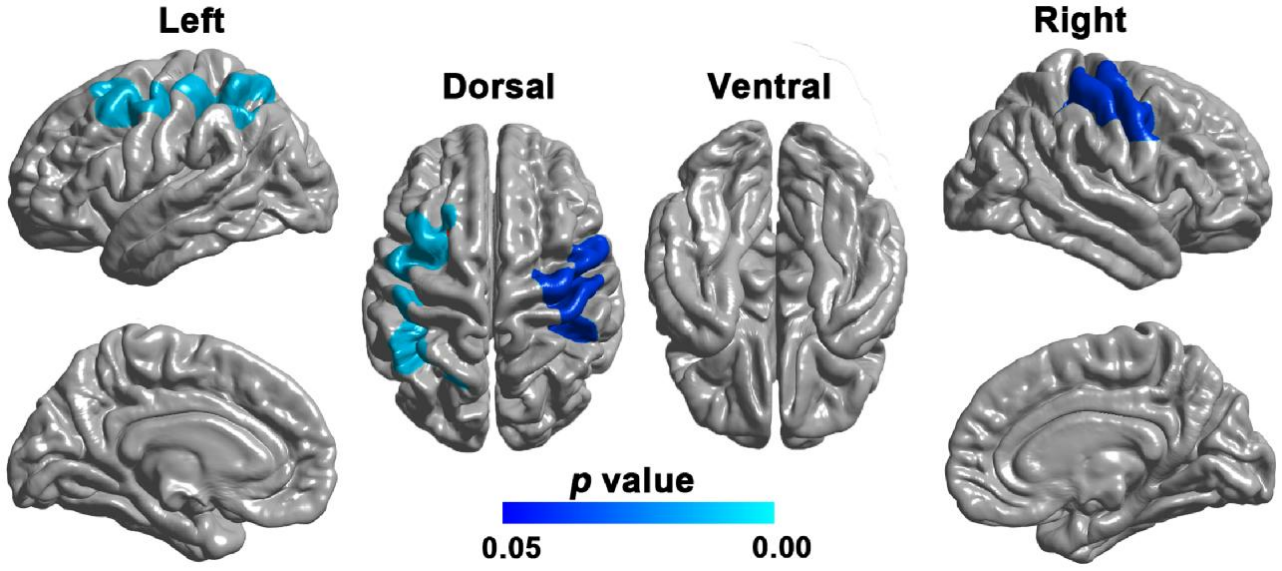
**Cortical regions showing significantly decreased LGI in patients with ALS (*N* = 60) compared with HCs (*N* = 60).** Statistical maps were generated using a vertex-wise GLM, controlling for age and sex. The results were thresholded at RFT-corrected (*P* < 0.05). The scale bar denotes the RFT-corrected *P* value.

Compared with HCs, patients with ALS showed decreased LGI in bilateral precentral and postcentral gyrus, left middle frontal gyrus and left superior parietal lobule (cluster size = 12987 vertices, peak Talairach coordinates: *x* = −21.61, *y* = 11.30, *z* = 51.65, peak *t*-value = 3.97 for the cluster on the left hemisphere; cluster size = 10,279 vertices, peak Talairach coordinates: *x* = 30.36, *y* = −12.79, *z* = 61.94, peak *t*-value = 3.18 for the cluster on the right hemisphere) (RFT-corrected *P* < 0.05; Fig. 1).

### Voxel-wise FA analysis

**Figure 2:**
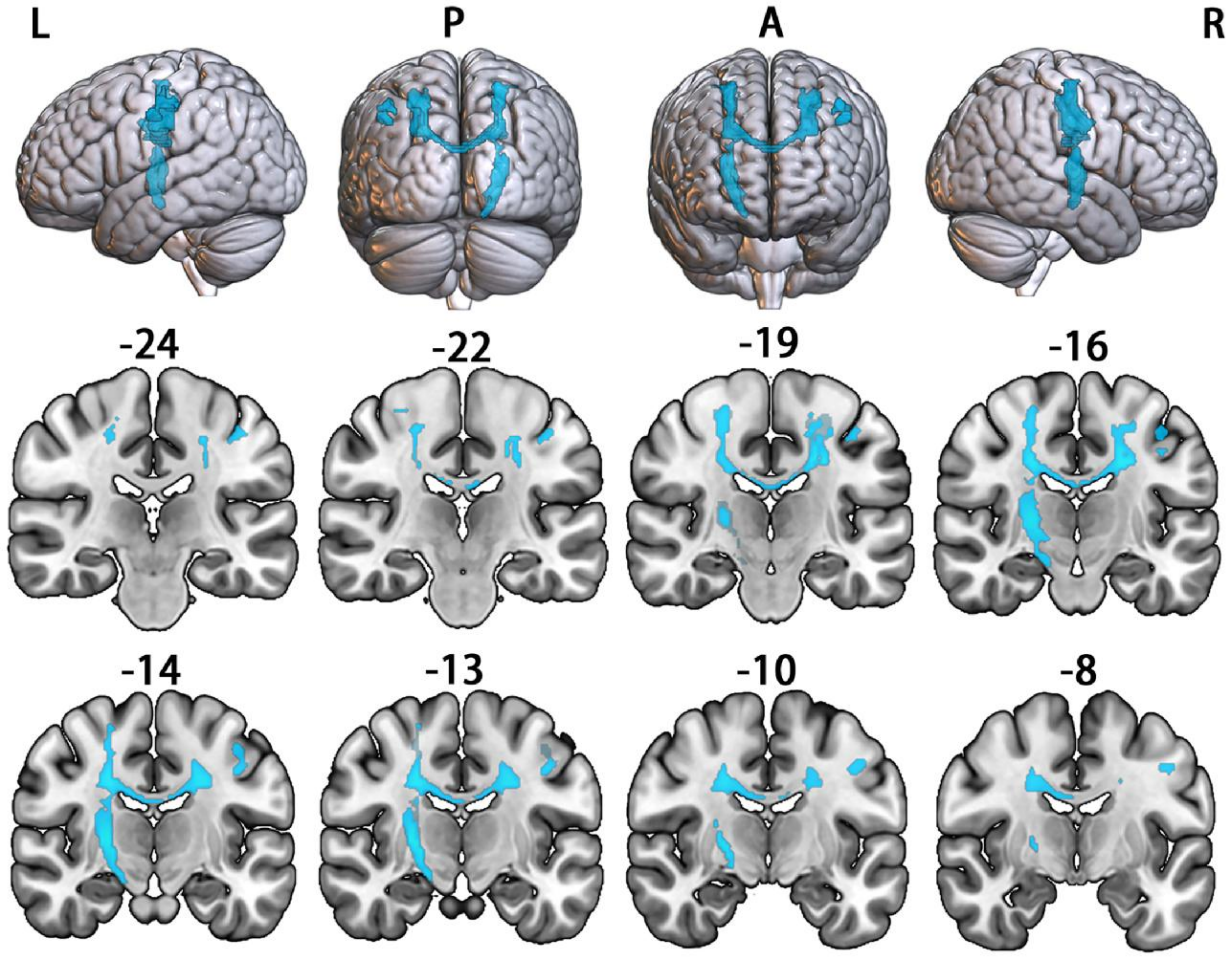
**Brain regions showing significantly decreased FA in patients with ALS (*N* = 60) compared with HCs (*N* = 60).** Statistical maps were generated using a voxel-wise GLM, controlling for age and sex. The results were thresholded at GRFT-corrected (*P* < 0.05). The highlighted region indicates the significant cluster. Numbers represent *y*-coordinates in MNI space. L = left; R = right; P = posterior; A = anterior.

Compared with HCs, voxel-wise contrasts of FA within the identified tracts of interest revealed one cluster where patients with ALS had significantly decreased FA (Fig. 2). This cluster mainly involved the bilateral corticospinal tract and the middle posterior body of the corpus callosum according to the JHU white matter atlas (Supplementary Fig. 2).

### Correlation analyses between imaging and clinical data

**Figure 3:**
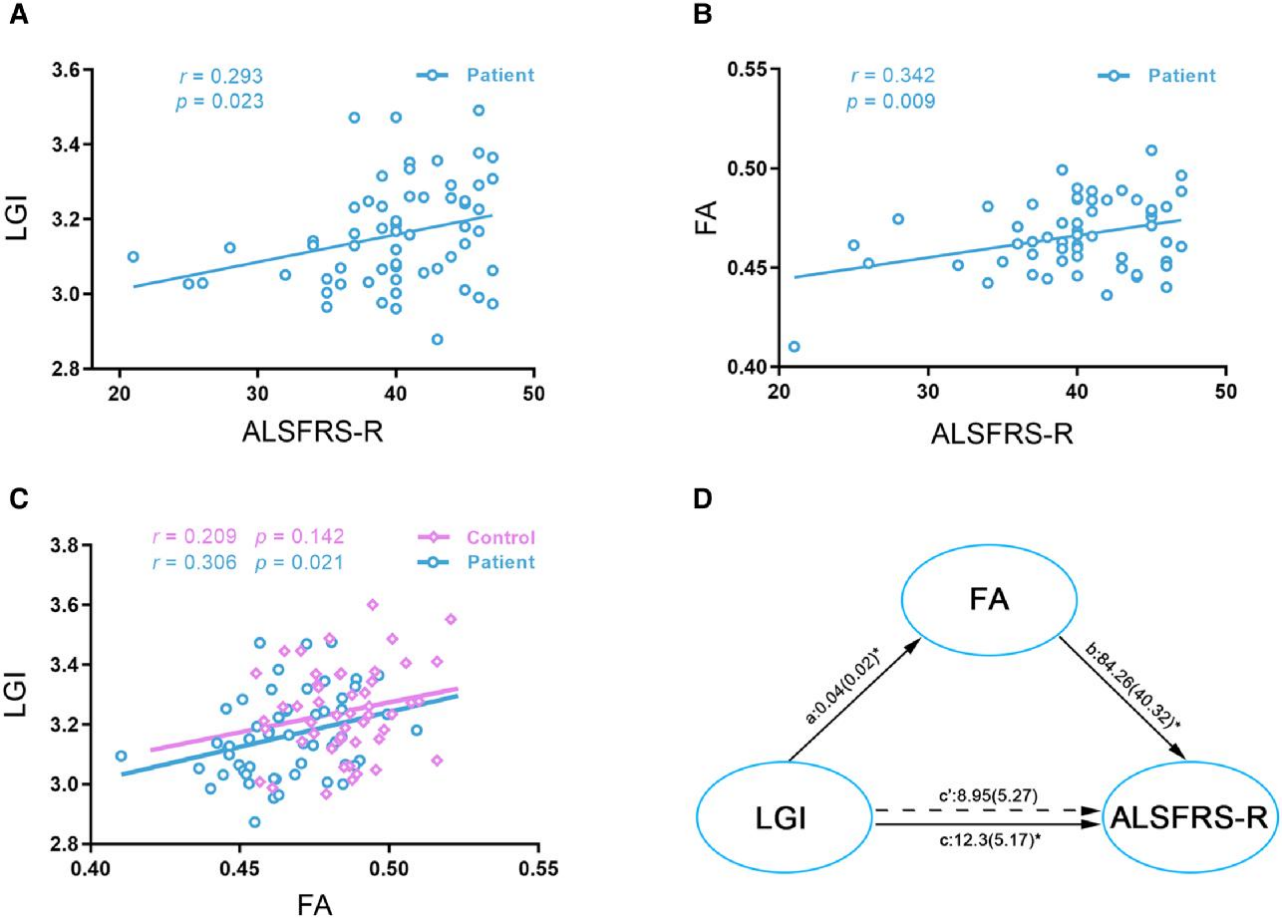
**Associations among LGI, FA and ALSFRS-R score in patients with ALS.** Panels **A**, **B**, and **C** display scatterplots based on Pearson’s correlation, where each data point represents an individual participant. (**A**) Positive correlation between LGI of the affected cortical regions and ALSFRS-R score. (**B**) Positive correlation between FA of the affected white matter tracts and ALSFRS-R score. (**C**) Positive correlation between the mean LGI of the affected cortical regions and the mean FA of the affected white matter tracts in patients with ALS. (**D**) Mediation analysis showing that FA of the affected white matter tracts fully mediates the relationship between LGI of the affected cortical regions and the ALSFRS-R score in patients with ALS. Path coefficients are shown with their standard errors in parentheses. This mediation model illustrates the direct effect of LGI on FA (Path a), the direct effect of FA on the ALSFRS-R score (Path b), the significant total effect of LGI on the ALSFRS-R score (Path c) and the non-significant direct effect of LGI on the ALSFRS-R score after controlling for FA (Path c′). A full mediation is demonstrated, as the significant total effect (Path **c**) is rendered non-significant (Path c′) by the mediator, and the indirect effect (Path a * Path b) is significant (*P* < 0.05). * indicates *P* < 0.05, two-tailed.

Higher mean LGI of the significant clusters related to higher ALSFRS-R (*r* = 0.293 and *P* = 0.023) (Fig. 3A) and MMSE (*r* = 0.340 and *P* = 0.008) scores. Higher mean FA of the significant clusters related to higher ALSFRS-R score in patients with ALS (*r* = 0.342, *P* = 0.009) (Fig. 3B). There was no significant correlation between the mean LGI/FA of the significant clusters and other clinical variables, including disease duration, HAMD, HAMA and FSS scores (all *P*-values > 0.05).

### Intermodal correlation analysis

Higher mean LGI of the affected cortical regions related to higher mean FA of the affected white matter tracts in patients with ALS (*r* = 0.306 and *P* = 0.021), whereas such relationship was not found in HCs (*r* = 0.209 and *P* = 0.142) (Fig. 3C).

### Mediation analyses

In patients with ALS, the FA of the affected white matter tracts had a significant mediating effect on the relationship between LGI of the affected cortical regions and the ALSFRS-R score, whereas the LGI of the affected cortical regions was not a significant mediator of the relationship between the FA of affected white matter tracts and the ALSFRS-R score (Fig. 3D).

### Neuroimaging–transcription association analysis

**Figure 4:**
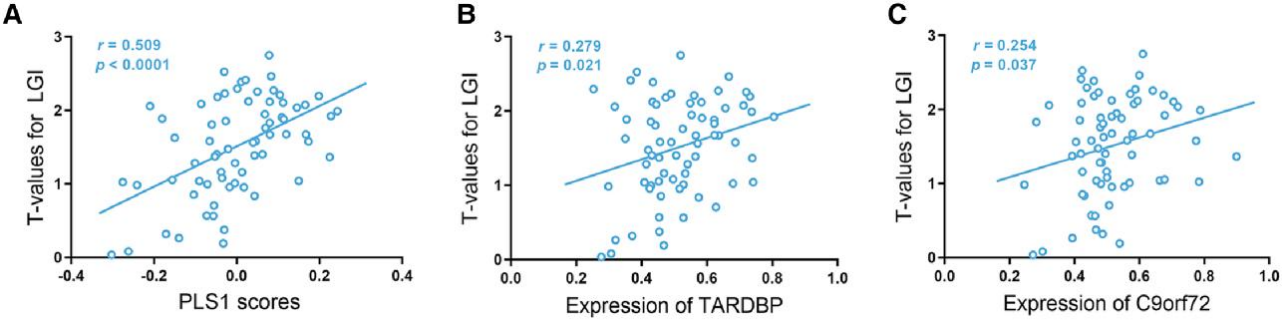
**Associations of *t*-values of regional LGI deviance with regional expression data. All panels display scatterplots based on Pearson’s correlation, with each data point corresponding to one of the 68 cortical regions.** (**A**) Positive correlation between regional PLS1 scores and the *t*-values for regional LGI deviance. (**B**) Positive correlation between regional expression of *TARDBP* and the *t*-values for regional LGI deviance. (**C**) Positive correlation between regional expression of *C9orf72* and the *t*-values for regional LGI deviance.

**Figure 5:**
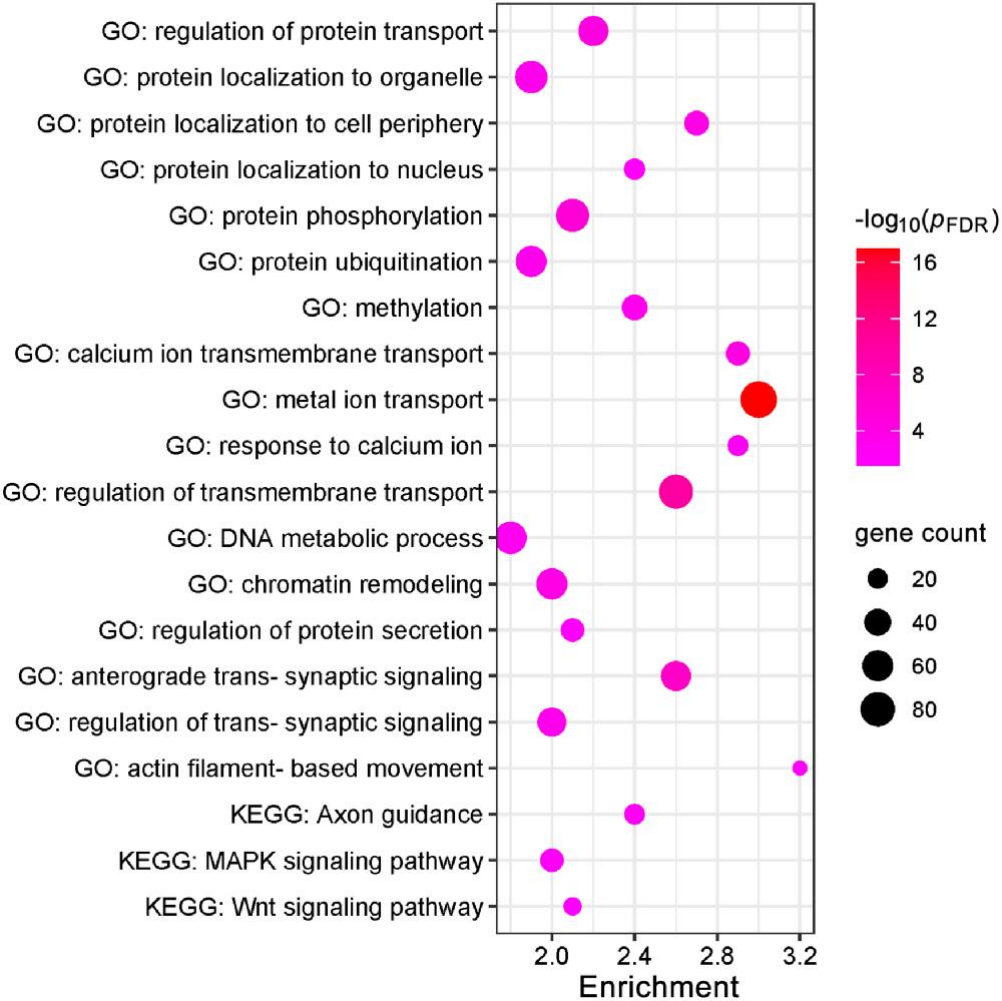
**Functional enrichment analysis of the pivotal genes associated with lower LGI in patients with ALS (*N* = 60).** Terms were retained with a threshold of false discovery rate (FDR) corrected *P* < 0.05.

The regional PLS1 scores calculated as the weighted sum of the 16,533 gene expression data was significantly correlated with the *t*-values of regional LGI deviance between the two groups (Fig. 4A), accounting for 25.9% of the variance of the statistical contrast (*r* = 0.509, permuted *P* = 0.030). With a threshold of *Z*-score > 3, we identified a total of 1498 pivotal genes associated with lower LGI in patients with ALS, including *TARDBP* (TDP-43 encoding gene), *C9orf72*, among others. Further bivariate correlation analyses using Pearson’s correlation coefficient confirmed the associations of *t*-values for regional LGI deviance with regional expression data of *TARDBP* (Fig. 4B) and *C9orf72* (Fig. 4C). Using the identified gene list, functional enrichment analysis revealed a number of biological processes/pathways highly relevant to the formation of pTDP43 aggregates (such as ‘regulation of protein transport’, ‘protein localization to nucleus’, ‘protein phosphorylation’, ‘calcium ion transmembrane transport’, ‘metal ion transport’, among others) and their propagation along axonal pathways (such as ‘axon guidance’, ‘MAPK signalling pathway’ and ‘Wnt signalling pathway’) (Fig. 5).

## Discussion

The present study examined cortical gyrification changes as well as their microstructural and transcriptional basis in patients with ALS. Primary findings of our study were summarized as follows. First, compared with HCs, patients with ALS showed significantly reduced LGI in the bilateral precentral and postcentral gyrus, left middle frontal gyrus and left superior parietal lobule, and the mean LGI of these regions was positively correlated with the ALSFRS-R score. Second, patients with ALS had significantly reduced FA in the white matter tracts connected to cortical regions with atypical gyrification, involving bilateral corticospinal tract and corpus callosum, and the mean FA of the affected white matter tracts was positively correlated with ALSFRS-R score. Third, the mean LGI of the affected cortical regions was positively correlated with the mean FA of the affected white matter tracts in patients with ALS. Fourth, the FA of the affected white matter tracts had a significant mediating effect on the relationship between LGI of the affected cortical regions and the ALSFRS-R score. Last, the atypical gyrification in ALS was associated with biological processes/pathways critically involved in the formation of pTDP-43 aggregates and their propagation along axonal pathways.

Compared with HCs, patients with ALS showed significantly decreased LGI in the bilateral precentral and postcentral gyrus, left middle frontal gyrus and superior parietal lobule. These results were partially supported by previous studies, which reported decreased gyrification in similar regions in asymptomatic carriers with a higher genetic risk of ALS as compared to control subjects.^18,19^ Nevertheless, a preliminary study examining a sample of 25 symptomatic patients with ALS found significantly decreased gyrification in the visual cortex,^20^ which appears to be at odds with current findings. The exact mechanism underlying such inconsistency however remains unknown and may arise from sample heterogeneities across studies. Of note, the cortical regions with atypical gyrification in this study corresponded well with Braak’s four-stage model of ALS based on the regional distribution of pTDP-43, involving the primary motor cortex in stage 1, and the premotor and somatosensory cortices in stage 2.^7,8^ This observation fits well with the clinical profile of our cohort, which was dominated by early-stage patients (median disease duration of 12 months; 75% at King’s Stage 1 or 2). Our present results, together with those of earlier presymptomatic studies, indicate that the LGI reductions likely relate to the stereotypic propagation of pTDP-43 and may have contributed to the progression of the motor impairments in these patients. Indeed, the precentral gyrus plays a vital role in the execution of voluntary motor movements.^31,32^ The middle frontal gyrus, also known as the premotor cortex, has been crucially involved in the preparation, execution and awareness of movement.^33,34^ In addition to motor functions, the middle frontal gyrus also plays roles in various higher cognitive functions such as attention, working memory, cognitive flexibility and so on.^15,35-37^ The postcentral gyrus, being the key region of the primary somatosensory cortex, is responsible for processing somatosensory information and the integration of sensory and motor signals.^38^ Our speculation about the involvement of LGI reductions in motor impairment was further supported by the finding of a significant positive correlation between the mean LGI of affected regions and ALSFRS-R score in these patients. In addition, we also note lower mean LGI in patients with a lower MMSE score despite the patient group not being selected for cognitive impairment or late stages of illness. This crucial relationship indicates that the change in cortical folding in motor regions is a reflection of a ‘spreading’ pathology, which while not yet apparent in other regions, is indicative of the impending progression. The observed correlation between LGI and ALSFRS-R score is thus consistent with the LGI—MMSE relationship highlighting the ability of gyrification index to track both functional and cognitive dysfunctions in ALS.^39-41^

Compared with HCs, patients with ALS showed significantly decreased FA in the white matter tracts connected to regions with atypical gyrification, mainly involving the bilateral corticospinal tract and middle posterior body of the corpus callosum. These microstructural changes are among the most consistently reported findings on the white matter abnormalities in patients with ALS and have thus been considered to be the characteristic pathologic features of this disorder.^42,43^ The corticospinal tract, originating mainly from the primary motor cortex, premotor area and somatosensory cortex,^44^ plays a crucial role in supporting voluntary distal movements.^45^ The middle posterior body of the corpus callosum connects the bilateral primary motor cortices on the two hemispheres and has been implicated in diverse motor functions such as bimanual coordination and fine motor control.^46-48^ Hence, the observed FA reductions in these tracts may be implicated in the motor impairments of patients with ALS, in keeping with our finding of a significant positive correlation between the mean FA and ALSFRS-R score in these patients. In addition, the mean FA of these white matter tracts was found to correlate with the mean LGI of the affected cortical regions in patients with ALS, indicating that changes in these two features are not independent. This result likely suggests that the microstructural abnormalities of the corticospinal tract and the corpus callosum play a role in the atypical gyrification in ALS. This speculation is consistent with the tension-based cortical morphogenesis, which posits that cortical folding is mainly driven by the tension of underlying axonal connections.^14^ More interestingly, we revealed a significant mediating effect of FA on the relationship between LGI and ALSFRS-R score in patients with ALS. The mediated relationship among the three variables indicates that the effects of gyrification reductions on motor functions in patients with ALS are exerted through disrupted white matter microstructure in tracts connected to the affected cortical regions. This finding has important implications for our understanding of the grey and white matter changes associated with ALS and how they underlie the motor impairments of this disorder.

In addition, PLS regression analysis was conducted to determine the expression profiles of the genes highly related to the LGI reductions, and we identified a total of 1498 pivotal genes, including *C9orf72*, *TARDBP*, among others. In line with the PLS regression analysis, bivariate correlation analysis revealed a significant positive correlation between regional expression of *TARDBP* and *t*-values for regional LGI deviance, indicating a crucial role of TDP-43 proteinopathies in the gyrification reductions in ALS. More intriguingly, the identified pivotal genes were significantly enriched in GO processes that are supportive of either a toxic gain or loss of TDP-43 function. Specifically, dysregulations in protein transport (such as nucleocytoplasmic transport)^49^ and protein localization are key drivers of cytoplasmic mislocalization and accumulation of the predominately nuclear RNA-binding TDP-43.^50^ Post-translational modifications of TDP-43, such as phosphorylation and ubiquitination, could have an effect on its activity, stability, cellular localization and aggregation propensity.^51^ Changes in cytosolic calcium are also known to play a role in regulating cytoplasmic accumulation of TDP-43.^52^ This connection is further substantiated by our finding of a core set of hub genes shared between the ‘metal ion transport’ and ‘regulation of protein transport’ pathways. Crucially, these genes (e.g. ITPR1 and SNAP25) are key regulators of calcium-dependent synaptic vesicle exocytosis,^53,54^ mechanistically linking dysregulated calcium homeostasis with defects in protein transport and release.^55,56^ All these processes may potentially contribute to the formation of pTDP-43 aggregates that are present in approximately 97% of patients with ALS.^52,57^ Meanwhile, TDP-43 plays a key role in DNA double-strand break repair^58^; a loss of nuclear TDP-43 has been linked to chromatin remodelling and DNA damage.^58-61^ In addition, the identified set of genes were also significantly enriched in KEGG pathways including axon guidance, Wnt and MAPK signalling pathways, all of which are involved in the development, maintenance and/or degeneration of axonal connections,^62-64^ and have been found to be affected in ALS.^57,62^ Moreover, there is also evidence supporting a role in axonal transport for Wnt and MAPK signalling pathways,^65,66^ which appear to interact with TDP-43 pathology and contribute to the white matter dysconnectivity in ALS.^64,67-69^ In summary, our results indicate that the gyrification reductions in ALS may be a macroscopic manifestation of the abnormal biological processes or pathways critically involved in formation of pTDP-43 aggregates as well as their propagation along axonal pathways.^7,14,70,71^

This study has several limitations that should be acknowledged. First, the permutation testing procedure in the neuroimaging-transcriptional association analysis does not explicitly preserve the spatial autocorrelation structure present in the transcriptomic and imaging data. While this approach is widely used and offers interpretability, it may inflate the risk of false positives in the context of spatially smooth brain maps. To address this, we conducted a complementary analysis using spatially constrained null models based on spherical permutations.^72,73^ This more conservative method attenuated the statistical significance of the observed association, potentially reflecting reduced power when correcting for spatial autocorrelation (*P* = 0.451). These findings highlight the importance of choosing appropriate null models and interpreting neuroimaging–transcription associations in light of spatial dependencies. Second, the correlations observed between imaging metrics and clinical scores, while statistically significant, were not robust and did not survive a strict correction for multiple comparisons using false discovery rate method. This lack of a strong, corrected association is likely due to the study’s moderate sample size, and therefore, while our findings establish an important link between cortical morphology and clinical status, the utility of gyrification as a robust biomarker for individual patient monitoring requires confirmation in larger, longitudinal cohorts. Finally, the study’s cross-sectional design and unbalanced subgroups for onset type precluded analysis of dynamic changes or subtype-specific effects, underscoring the need for future longitudinal research in larger, more balanced patient populations.

In conclusion, we found significant gyrification reductions that were associated with white matter microstructural abnormalities and biological processes/pathways highly relevant to the formation and propagation of pTDP-43 aggregates in ALS. These findings may contribute imaging markers for ALS progression and potential pathways for early intervention of ALS.

## Supplementary Material

fcaf491_Supplementary_Data

## Data Availability

The data that support the findings of the present study are available from the corresponding authors upon reasonable request.
