# Supplementary material for: Microstructure and gene expression influence gyrification in amyotrophic lateral sclerosis
Source: Brain Commun. 2025 Dec 16;8(1):fcaf491. doi: 10.1093/braincomms/fcaf491 (PMC12782020; doi:10.1093/braincomms/fcaf491)
Supplement: fcaf491_Supplementary_Data [file fcaf491_supplementary_data.pdf]

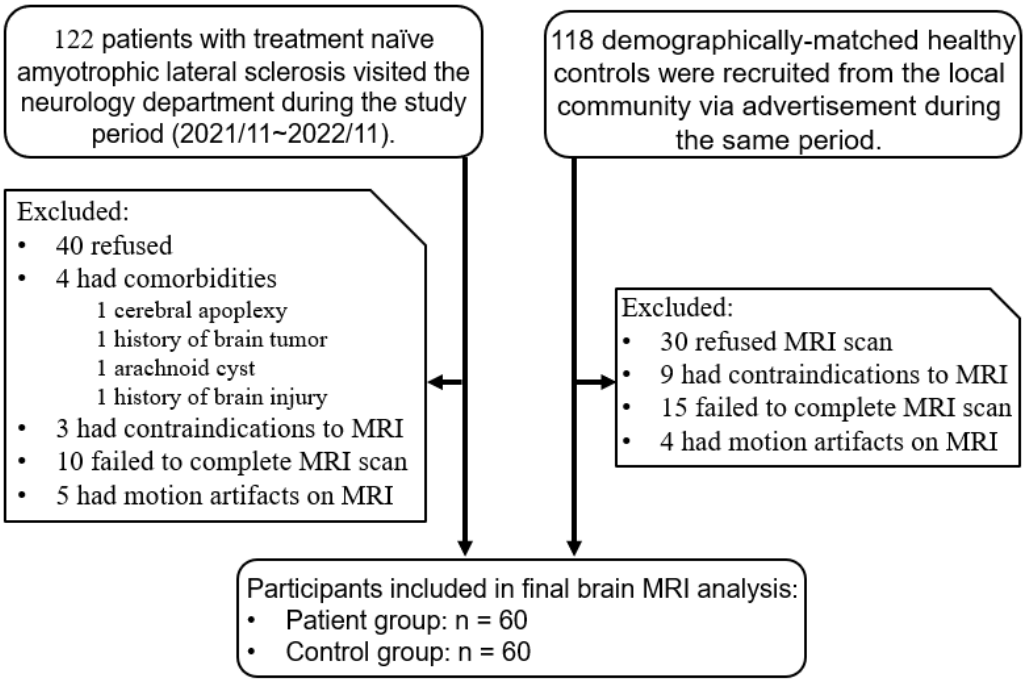

Supplementary Figure 1 Flowchart for the exclusion and inclusion of patients with Amyotrophic Lateral Sclerosis and healthy controls.

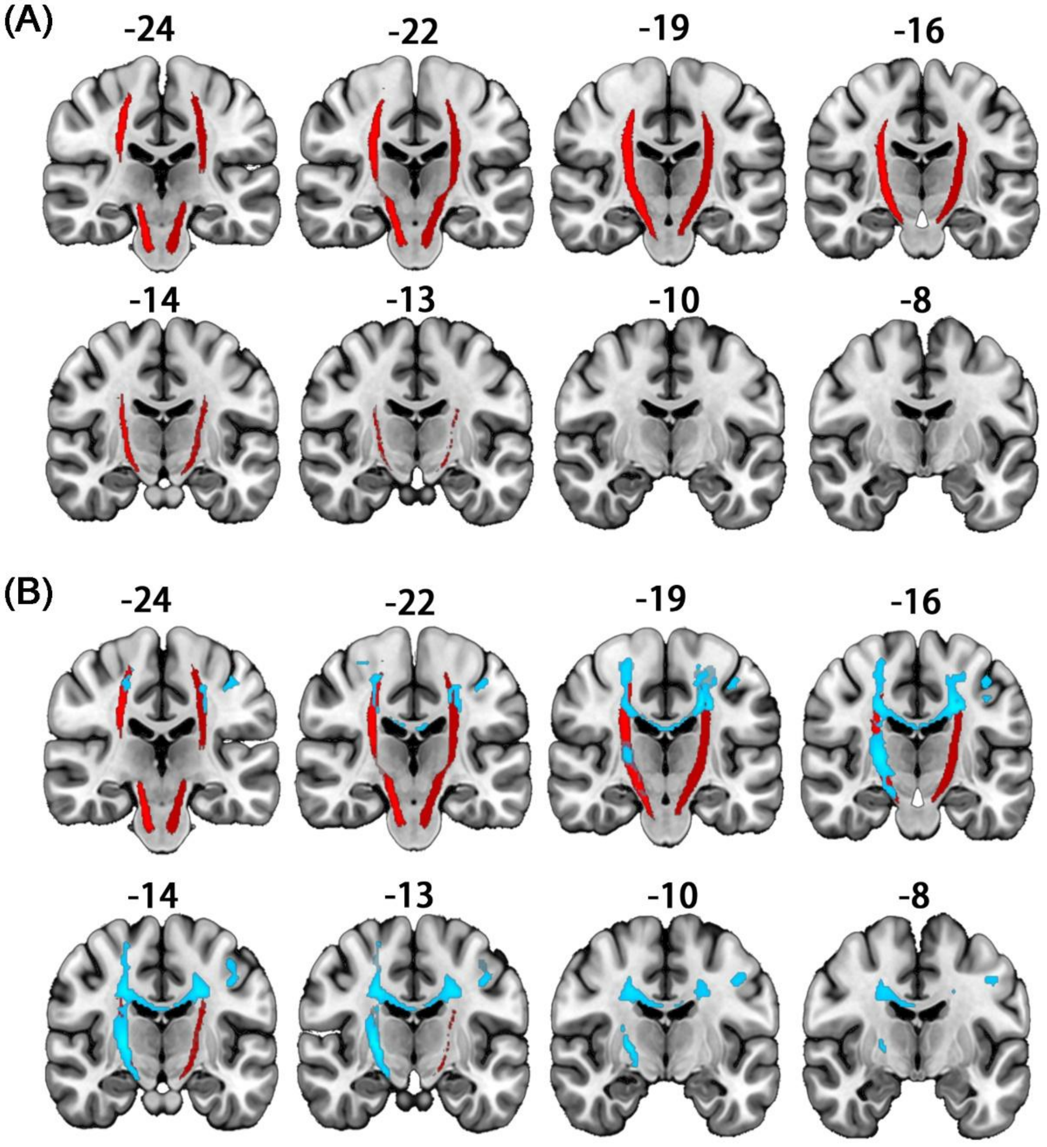

Supplementary Figure 2 Overlap of significant fractional anisotropy (FA) reduction with the corticospinal tract (CST). (A) The mask of the CST, shown in red, was derived from the JHU white matter atlas and is displayed for anatomical reference. (B) The cluster of significantly reduced FA in patients with Amyotrophic lateral sclerosis (blue) is shown overlaid on the CST mask (red).
